# Sulcatone as a Plant-Derived Volatile Organic Compound for the Control of the Maize Weevil and Its Associated Phytopathogenic Fungi in Stored Maize

**DOI:** 10.3390/plants13202893

**Published:** 2024-10-16

**Authors:** Fernanda Achimón, Maria L. Peschiutta, Vanessa D. Brito, Sofia B. Ulla, Romina P. Pizzolitto

**Affiliations:** 1 Instituto Multidisciplinario de Biología Vegetal (IMBIV-CONICET), Universidad Nacional de Córdoba, Av. Vélez Sarsfield 1611, Córdoba X5016GCA, Argentina; fachimon@imbiv.unc.edu.ar (F.A.); mlpeschiutta@imbiv.unc.edu.ar (M.L.P.); vbrito@imbiv.unc.edu.ar (V.D.B.); s.ulla@imbiv.unc.edu.ar (S.B.U.); 2 Instituto de Ciencia y Tecnología de los Alimentos (ICTA), FCEFyN, Universidad Nacional de Córdoba, Av. Vélez Sarsfield 1611, Córdoba X5016GCA, Argentina

**Keywords:** *Sitophilus zeamais*, insect vector, *Fusarium verticillioides*, *Aspergillus flavus*, *Aspergillus parasiticus*, sulcatone, volatile organic compounds, mycotoxins, biopesticide

## Abstract

Stored maize is frequently attacked by different pests, such as insects and microorganisms. The aim of the present study was to evaluate the bioactivities of sulcatone (6-methyl-5-hepten-2-one) against the maize weevil *Sitophilus zeamais* and the phytopathogenic fungi *Fusarium verticillioides*, *Aspergillus flavus*, and *A. parasiticus.* Sulcatone showed a strong repellent effect with a maximum value of −92.1 ± 3.2% at 40 µM in two-choice olfactometer bioassays and an LC_95_ value of 17.2 µL/L air (95% 16.5–18.1) in a fumigant toxicity experiment. The antifungal effect of sulcatone was evaluated through the fumigant method, reporting MIC values of 3.5, 3.8, and 3.9 mM for *F. verticillioides*, *A. parasiticus*, and *A. flavus*, respectively. Additionally, a silo-bag experiment containing all pests was conducted to evaluate the potential use of sulcatone in a real storage system. Sulcatone caused 71.69 ± 1.57% weevil mortality in silo-bags and proved to be effective as a fungicidal and antimycotoxigenic agent since both ergosterol and fumonisin B_1_ content were significantly reduced by 60% in silo-bags containing sulcatone. This study demonstrated that sulcatone has the potential to be used for the control of both insects and fungi of stored maize, without affecting the germination of grains.

## 1. Introduction 

**Figure 1:**
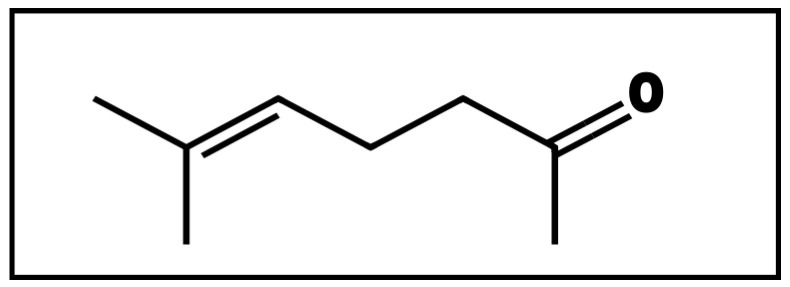
Molecular structure of sulcatone (6-methyl-5-hepten-2-one).

Maize is one of the world’s leading crops, widely cultivated as a cereal grain and an important source of nutrients, minerals, and vitamins for both human and animal diets. After harvest, maize grains are temporarily preserved in storage systems, such as trench silos or silo-bags [1]. *Sitophilus zeamais* Motschulsky (Colepotera; Curculionidae) is considered the major insect pest of stored maize due to the severe damage caused to grains through its reproductive and feeding habits [2]. The activity of maize weevils reduces grain weight and nutrient content, causing huge economic losses. Additionally, the respiratory activity of insects increases the humidity and temperature of silos, favoring the proliferation of filamentous fungi [3,4]. In this context, the prevailing fungal species associated with stored maize are *Fusarium verticillioides*, *Aspergillus flavus*, and *Aspergillus parasiticus* [1,4]. The growth and metabolic activity of these fungi leads to the deterioration of the physical, nutritional, and organoleptic qualities of grains [5]. Furthermore, these fungal species are prolific producers of mycotoxins, such as fumonisins and aflatoxins, which are reported as highly toxic to humans and farm animals [6,7,8]. One of the most important mechanisms of fungal contamination on maize is through the activity of insect vectors since the constant movement of weevils inside the silo contributes to the dispersal of fungal spores, hyphae, and mycotoxins from contaminated to uncontaminated grains [9]. The control of the maize weevil and its associated phytopathogenic fungi currently depends on the application of synthetic pesticides, specifically insecticides and fungicides. Despite the efficacy of these compounds, their frequent application has been associated with hazardous effects on living organisms and the environment [10,11]. In this context, certain natural compounds, such as plant essential oils (EOs) and their pure volatile organic compounds (VOCs), have been proposed as environmentally friendly alternatives to synthetic pesticides [12,13]. Essential oils are complex mixtures of different classes of VOCs, of which terpene hydrocarbons and their oxygenated derivatives usually predominate. Among oxygenated terpenes, ketones have been proposed as highly reactive VOCs with important pesticidal properties [14,15]. In this context, the unsaturated methyl ketone known as sulcatone (6-methyl-5-hepten-2-one; Figure 1) is synthesized by more than 400 plant species, being the major constituent of Citronella, Lemon-grass, and Palm Rose EOs [16,17,18,19,20,21,22,23]. 

According to the aforementioned, the aims of the present study were to: (i) evaluate the repellent and insecticidal effects of sulcatone against *S. zeamais* and (ii) determine the fungicidal and antimycotoxigenic effects of sulcatone against *F. verticillioides*, *A. flavus*, and *A*. *parasiticus*. Since these organisms interact in stored maize, a silo-bag experiment including weevils and the three phytopathogenic fungi was conducted. The results obtained here will allow exploration of sulcatone to be used for the integrated control of maize pests to further establish a basis for the development of new biopesticides.

## 2. Materials and Methods

### 2.1. Reagents

Sulcatone (6-methyl-5-hepten-2-one; 99%; #CAS 110-93-0) and propionic acid (99.5%; #CAS 79-09-4) were purchased from Sigma-Aldrich (Buenos Aires, Argentina). 

### 2.2. Insect Rearing

Unsexed adults of *S. zeamais* reared on free-insecticide maize whole grains under controlled conditions of temperature and relative humidity (27 ± 1 °C and 65 ± 2% RH) were used. All the experiments were conducted in complete darkness and controlled environmental conditions. 

### 2.3. Fungal Strains and Inoculum Preparation

The fungal strains *F. verticillioides* M3125, *A. parasiticus* NRRL 2999, and *A. flavus* NRRL 3251 were used. *Fusarium verticillioides* M3125 was provided by Dr. Robert Proctor (United States Department of Agriculture, Agricultural Research Service, National Center for Agricultural Utilization Research, Peoria, IL, USA) and is a prolific producer of fumonisins. *Aspergillus* sp. strains were obtained from the Mycology Reference Center (National University of Rosario; Santa Fe, Argentina). For each fungal species, inoculum was prepared by adding sterile distilled water to 10-day-old cultures grown on Czapek Dox Agar (CDA; Oxoid) at 25 °C in the dark. The conidial final concentration was adjusted to 1 × 10^6^ conidia/mL using a Neubauer chamber (Marienfeld, Germany). 

### 2.4. Repellency Assays

To evaluate the behavioral response of *S. zeamais* to sulcatone, a two-choice olfactometer bioassay was conducted according to Herrera et al. [24] with some modifications. Briefly, two flasks of 100 mL were connected to a glass tube (30 cm long, 1 cm diameter) with a central hole of (1 cm^2^). A filter paper of 1 cm^2^ was placed within each flask, and sulcatone was added to the filter paper of only one flask (treatment), while the other remained odorless (control). The concentrations evaluated were 0.4, 4.0, and 40.0 µM. A total of 20 insects were released in the central hole of the glass tube, and the olfactometer was placed for 2 h in a climatic chamber at 27 ± 1 °C and 65 ± 2% RH. The position of the flasks was changed at every replicate, and propionic was used as the repellent control. In each test, a response index (RI) was calculated using the following equation: *RI* = [(*T* − *C*)/*Tot*] × 100, where *T* is the number of insects that respond to the treatment, *C* is the number of insects that respond to the control, and *Tot* is the total number of insects released. Positive values of RI indicate attraction to the treatment, while negative values indicate repellency [24]. Those insects that showed no response in the olfactometer were not considered in the calculation of the RI. Independent controls consisting of two vials without the addition of any compound were conducted to determine whether the movement of the insects toward the flasks was random in the absence of any VOC. The experiment was conducted three times per concentration with five replicates each.

### 2.5. Fumigant Toxicity Bioassay

To assess the fumigant toxicity of sulcatone, ten adults of *S. zeamais* were placed in 100 mL glass vials sealed with rubber stoppers. Different amounts of sulcatone were included in a 4 cm^2^ filter paper hanging from the top of the vial, and the concentrations tested ranged from 5 to 20 µL/L. Filter papers without sulcatone were used as controls. The glass vials were placed in a rearing chamber at 27 ± 1 °C and 65 ± 2% RH, and mortality of insects was determined after 24 h of exposure. Five replicates were performed for each concentration, and the experiment was repeated twice.

### 2.6. Determination of Minimum Inhibitory Concentrations (MIC) 

The inhibitory activity of sulcatone was determined separately for *F. verticillioides*, *A. parasiticus*, and *A. flavus* using maize meal extract agar (MMEA; 30 g/L maize meal and 20 g/L agar) through the fumigant methodology described by Achimón and Pizzolitto [25]. A 90 mm filter paper was placed on the inside cover of the MMEA Petri plate, and sulcatone was added to the filter paper at 0.03, 0.06, 0.13, 0.27, 0.53, 1.06, 2.12, and 4.24 mM. Filter papers without sulcatone were used in the control Petri plates. Then, 10 µL of the conidial suspension (1 × 10^6^ conidia/mL) was centrally inoculated in each Petri plate and incubated at 25 °C in the dark. The diameters of the treatment colonies were measured when the control colonies reached the edge of the plate (on day 8 for *F. verticillioides* and on day 10 for *A. flavus* and *A. parasiticus*). The inhibition percentage was plotted against sulcatone concentration, and a linear regression was applied (*y = a + bx)* to obtain the intercept (a) and the slope of the line (b). The MIC was defined as the lowest concentration of the compound at which no fungal growth was observed and was calculated for each fungal species using the formula *MIC* = (100 − *a*)/*b* [14]. Five replicates were performed for each concentration, and the experiment was repeated twice.

### 2.7. Silo-Bag Experiment

To unveil the potential role of *S. zeamais* as an insect vector of phytopathogenic fungi and to study the ability of sulcatone to control the different maize pests, a silo-bag experiment was conducted. Sterilized silo bags of 170 mL containing 60 g of maize were used [26], and the following treatments were defined: (i) *F. verticillioides* + *A. flavus* + *A. parasiticus*; (ii) *F. verticillioides* + *A. flavus* + *A. parasiticus* + *S. zeamais*; (iii) *F. verticillioides* + *A. flavus* + *A. parasiticus* + sulcatone; (iv) *F. verticillioides* + *A. flavus* + *A. parasiticus* + *S. zeamais* + sulcatone; (v) *S. zeamais*; (vi) *S. zeamais* + sulcatone; and (vii) control (maize). For those treatments containing fungi, the silo-bags were inoculated with 500 µL of the conidial suspension of each fungus (1 × 10^6^ conidia/mL). A total of 30 insects were added to the silo-bags of treatments containing insects. In treatments containing sulcatone, the VOC was included in a filter paper of 8 cm^2^ and placed inside the silo-bags at a concentration corresponding to twice the maximum MIC value (2 × MIC). The silo-bags were placed in a rearing chamber at 27 ± 1 °C and 65 ± 2% RH for 30 days. After the incubation period, the following parameters were determined: amount of ergosterol (µg ergosterol/ g maize), production of fumonisin B_1_ (µg FB_1_/ µg ergosterol), and mortality of insects (%). After the quantification of dead insects, silo-bags were autoclaved and left to dry in an oven at 60 °C for 10 days for ergosterol and fumonisin B_1_ determinations. Five replicates were performed for each treatment, and the experiment was repeated twice.

### 2.8. Ergosterol Extraction and Quantification

Ergosterol is the main cell membrane sterol specific to fungi that is commonly used as an indicator of fungal biomass. The amount of ergosterol was quantified to determine fungal growth in silo-bags. The extraction and quantification of ergosterol was conducted following the methodology proposed by Young [27] with some modifications. Briefly, the dried content of each silo-bag was ground to a fine dust. A sample of 100 mg was suspended in a solution containing 2 mL of methanol and 0.5 mL of sodium hydroxide (2 M), and the test tube was then sealed with a screw cap coated with Teflon. The test tubes were placed inside 500 mL plastic bottles screwed and sealed. The bottles were heated twice in a microwave oven (750 W) to 50% power for 20 s. Then, samples were left to cool to room temperature, and 2 mL of n-hexane was added to the test tubes. The tube was vortexed for 20 s, and n-hexane was removed. This procedure was repeated once more, and a final volume of 4 mL of n-hexane was recovered. The n-hexane extracts were evaporated to dryness and then resuspended in 1 mL of methanol before its quantification by high-pressure liquid chromatography (HPLC; Perkin Elmer, MA, USA). A C18 reverse phase column (internal diameter of 150 mm × 4.6 mm and particle size of 5 µm; Phenomenex, CA, USA) connected to a C18 precolumn (internal diameter of 20 mm × 4.6 mm and particle size of 5 μm; Phenomenex, CA, USA) was used. The mobile phase was acetonitrile–methanol (80:20), and the flow rate was 1.3 mL/min. The quantification of ergosterol was carried out by comparing sample peak areas with those from the analytical standards (Sigma-Aldrich, Buenos Aires, Argentina). 

### 2.9. Fumonisin B_1_ Extraction and Quantification

The extraction and quantification of fumonisin B_1_ was performed following the methodology of Brito et al. [28]. Samples of 10 g of grounded maize were mixed with 30 mL of distilled water, shaken at 200 rpm in a rotary shaker for 2 h, and then centrifuged at 5000 rpm for 10 min. Subsequently, 1 mL of the supernatant was centrifuged at 14,000 rpm for 15 min. An aliquot of 500 µL was mixed with 500 µL of acetonitrile. The quantification of fumonisin B_1_ was performed following the methodology proposed by Shephard et al. [29]. Previous to the injection, samples were derivatized for 3.3 min as follows: 50 µL of the samples + 200 µL of the derivatizing solution (40 mg orthophthaldehyde, 5 mL of sodium tetraborate 0.1 M + 50 μL of 2-mercaptoethanol + 1 mL of methanol). The samples were analyzed by Perkin Elmer HPLC equipped with a fluorescence detector. The wavelengths used were 335 nm for excitation and 440 nm for emission. An analytical reversal phase column C18 (internal diameter of 150 mm × 4.6 mm and particle size of 5 µm) connected to a precolumn C18 (internal diameter of 20 mm × 4.6 mm and particle size of 5 μm) was used. The mobile phase was methanol and sodium phosphate monobasic 0.1 M (75:25); the pH was adjusted at 3.35 ± 0.2 with orthophosphoric acid; the flow rate was 1.5 mL/min. The quantification of the mycotoxin was performed by comparing the sample peak areas with those from the analytical standards (Sigma-Aldrich, Buenos Aires, Argentina). 

### 2.10. Seed Germination Bioassay

To rule out the toxic effect of sulcatone on maize grains, a seed germination bioassay was conducted. Maize grains were superficially disinfected before their exposure to sulcatone. Grains were immersed in sodium hypochlorite 3% P/V, placed in a rotary shaker at 200 rpm for 30 min, and then rinsed three times with sterile distilled water. Ten maize seeds were placed in the bottom of a 9 cm Petri plate lined with autoclaved filter paper moistened with 3 mL of sterile distilled water. An aluminum foil of 2 cm diameter containing a 4 cm^2^ filter paper impregnated with sulcatone was placed in the center of the Petri dish [24]. The doses evaluated were those corresponding to the lethal concentration 95 (LC_95_), the highest repellence dose tested on *S. zeamais* (40 µM), and twice the maximum MIC value (2 × MIC). Petri plates without the addition of sulcatone were used as a control. Petri plates were closed and sealed to prevent the diffusion of sulcatone into the surrounding space, and then placed in a growth chamber at 27 ± 1 °C and 65 ± 2% RH for 7 days. A seed was considered as germinated when the radicle length was ≥2 mm. The number of germinated seeds was determined daily, and the seed vigor was calculated for each treatment through the equation: *seed vigor* = ∑(*n d* ^−1^)*,* where *n* is the number of seeds germinated on each day and *d* is the number of days elapsed since the beginning of the assay [24]. Five replicates were conducted for each treatment, and the experiment was repeated twice.

### 2.11. Data Analyses

Statistical analyses were conducted using INFOSTAT software v. 2020 (Universidad Nacional de Córdoba, Córdoba, Argentina). Data from these studies were analyzed by one-way analysis of variance (ANOVA) followed by a Di Rienzo, Guzmán, Casanoves (DGC) test (*p* < 0.05) [30]. The normality of data was tested using the Shapiro–Wilk test. Lethal concentrations causing 50% (LC_50_) and 95% (LC_95_) mortality and Chi-square statistic values (X^2^) of the adequacy of fit (*p* < 0.05) were calculated and subjected to a Probit regression analysis using the SPSS Software v. 26.0.0.0 at a 95% confidence interval. 

## 3. Results and Discussion

**Figure 2:**
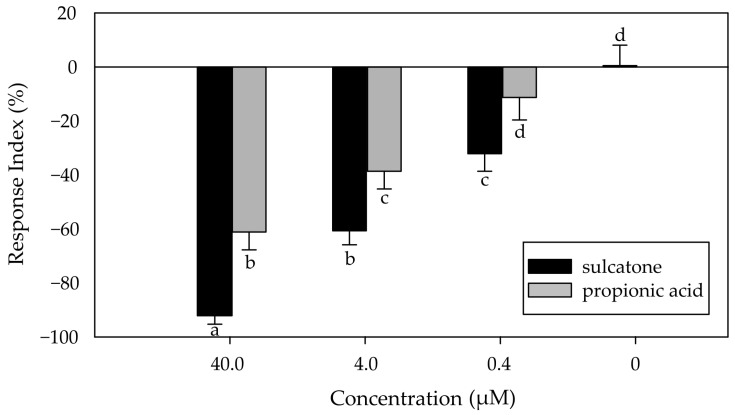
Response index of *S. zeamais* to sulcatone at 40.0, 4.0, and 0.4 µM. Negative values of RI indicate repellency. Black bars correspond to sulcatone; grey bars correspond to propionic acid (repellent control). Different letters indicate statistically significant differences between compounds and concentration according to a DGC a posteriori test (*p* < 0.05).

As can be seen in Figure 2, sulcatone was repellent to the maize weevil at all the concentrations tested, with RI values of −92.1 ± 3.2%, −60.7 ± 5.2%, and −32.1 ± 6.5% for 40 µM, 4 µM, and 0.4 µM, respectively. It is interesting to notice that the repellency caused by sulcatone was significantly higher compared to that of the repellent control, propionic acid, at all concentrations tested. For example, the highest RI of propionic acid was −61.2 ± 6.6% at 40 µM, comparable to that of sulcatone at a concentration 10 times lower. The repellent activity of natural VOCs against the maize weevil has been extensively studied [14,24,31,32,33]. Different phenolic compounds, alcohols, and ketones were reported as effective repellents to *S. zeamais* when evaluated using a two-choice olfactometer system. As mentioned before, sulcatone is an unsaturated aliphatic ketone, a hept-5-en-2-one substituted by a methyl group at position 6 (Figure 1). In this context, Pizzolitto et al. [14] evaluated the repellency of several ketones against the maize weevil, with thymoquinone and α-thujone being strong repellents with RI values of −77.8 and −75.5, respectively, at 4 µL/L, similar to that of propionic acid at the same concentration. Similarly, Herrera et al. [24] reported a RI of −91.2% at 4 µL/L for 3-octanone. Even though these studies expressed VOC concentration in a different way, 40 µM is equivalent to 5.96 µL/L for sulcatone, so results among studies can be compared since concentration values are similar. The results presented here are consistent with previous investigations that reported the repellent effect of sulcatone against different species of insects, such as ants, beetles, and aphids [34,35,36,37].

Regarding insecticidal activity, sulcatone reported a strong fumigant toxicity, with LC_50_ and LC_95_ values of 12.3 (CI 95% 11.8–12.7) and 17.2 (CI 95% 16.5–18.1) µL/L air, respectively, after 24 h of exposure (X^2^ = 20.3 at *p* < 0.05; slope 0.3 ± 0.03). Comparing these results with data from the literature, the LC_95_ value estimated in the present study was significantly lower than that obtained for other VOCs, such as alcohols, aldehydes, and long-carbon-chain linear ketones [24,31], indicating that sulcatone was more effective in controlling the maize weevil. According to current evidence, monoterpene ketones are more effective as insecticides than alcohols or hydrocarbons [38,39,40,41,42,43]. However, the degree of toxicity of different ketones depends on their molecular structure [44]. In this context, Herrera et al. [15] evaluated the insecticidal effect of several monoterpene ketones with different molecular structures and found that monocyclic structures were more bioactive compared to aliphatic and bicyclic structures, particularly thymoquinone and pulegone, which presented LC_95_ values of 19.2 and 14.2 µL/L air, respectively. Even though sulcatone is a linear ketone, it was as effective as thymoquinone and pulegone when applied as a fumigant. These results suggest that the molecular structure of a certain VOC affects different topological or physicochemical properties of the molecules, which ultimately determine their bioactivity. For example, the length of the carbon chain of methyl ketones exerts a significant effect on the polarity of VOCs (octanol-water partition coefficient; LogP), and thus on their bioactivity. This pattern was reported in a previous study evaluating the insecticidal activity of short-chain methyl ketones against *S. granarius*, which found that 2-pentanone (LogP 0.90) was more effective in killing adult weevils compared to 2-hexanone (LogP 1.44) and 2-heptanone (LogP 1.97) [45]. The insecticidal efficiency of VOCs depends on their ability to penetrate through the insect cuticle to reach their target sites, among other factors. The cuticle of insects consists of a lipophilic outer layer (the epicuticle) and a hydrophilic inner layer (the chitinous procuticle). The interaction between the thickness of the hydrophobic and hydrophilic layers and the polarity of a certain VOC determines its penetration capacity. Considering that the chitin fibers of the procuticle predominate in hard-bodied insects, such as weevils, hydrophilic VOCs (less hydrophobic, lower LogP) would penetrate easily [46]. In this context, sulcatone (LogP 2.09) is also a methyl ketone with higher hydrophilic properties compared to thymoquinone (LogP 2.33) or pulegone (LogP 2.56). It thus has a higher penetration capacity through the weevil cuticle, which could explain the strong insecticidal effect of sulcatone, regardless of its linear molecular structure. The insecticidal activity of sulcatone was reported against *Spodoptera littoralis*, which would be related to the inhibition of acetylcholinesterase activity [47]. 

Concerning the antifungal activity, sulcatone proved to be effective against the three phytopathogenic fungi in experiments conducted in Petri plates, since it reduced fungal growth in a dose-dependent manner. The three fungal species presented similar MIC values of 3.5, 3.8, and 3.9 mM for *F. verticillioides*, *A. parasiticus*, and *A. flavus*, respectively (Table 1). Ketones are VOCs characterized by a carbonyl functional group, consisting of a polarized carbon-oxygen double bond [48]. In general, ketones are considered reactive molecules: due to differences in their electronegativity, there is a partial positive charge on the carbon atom (electrophilic), which is prone to react easily with molecules with high electron density, such as amino acids and nucleic acids, affecting different fungal metabolic pathways [49]. However, as mentioned above, the bioactivity of VOCs depends on their specific molecular structure. In accordance with the present results, previous studies demonstrated the antifungal effect of different ketones against *F. verticillioides*. For example, the MIC value of the aliphatic methyl ketones 2-decanone, 3-decanone, and 3-octanone was ≈4.24 mM [24,31]. The bioactivity of these VOCs could be due to their long carbon chains that increase their lipophilicity, and hence their ability to cross the cell membrane lipid layers and reach the target molecules inside fungal cells [50]. A recent study reported the antifungal activity of the six-carbon ketones 3-hexanone (saturated) and 4-hexen-3-one (unsaturated) against *F. verticillioides*. These VOCs are structurally related and differ from each other due to the presence of an extra double bond in 4-hexen-3-one. This change in the molecular structure considerably affects their bioactivity, because 3-hexanone exhibited a growth stimulatory effect whereas 4-hexen-3-one showed strong inhibitory activity (0.3 mM) [25]. It is important to note that the double bond on 4-hexen-3-one is located between the α and β carbons (α,β-unsaturation), which increases the electrophilic properties of the molecule, being likely to react with thiol or amino groups of amino acids or nucleic acids [49]. Regarding monoterpene ketones, another study evaluated their antifungal effect against *F. verticillioides*, with thymoquinone and carvone being the most active compounds, which would be related to the α,β-unsaturations as well as the substituents at the β-carbon [14]. This is, to the best of our knowledge, the first report on the antifungal activity of sulcatone. Further research is required to explore the molecular mechanisms behind the antifungal activity of sulcatone. 

To evaluate the ability of sulcatone to control all the maize pests and the potential role of *S. zeamais* as a vector of phytopathogenic fungi, a silo-bag experiment including adult weevils and three fungal species was conducted. Table 2 displays the results from ergosterol content, fumonisin B_1_ production, and insect mortality percentage in maize silo-bags subjected to different treatments. As can be seen from the table, the amount of ergosterol was 42% higher when the maize weevil was present in treatments with fungi (but no sulcatone). These results are in agreement with previous studies that stated that the presence of insects may favor fungal infection of stored maize, either by damaging grains and making it more vulnerable to fungal attack, or by acting as fungal vectors [5,28,51,52,53]. For example, Ferreira-Castro et al. [51] reported the ability of weevils to disseminate fungal spores from contaminated maize grains to uncontaminated grains, with the subsequent production of mycotoxins. This effect was also observed in other ecological interactions in which the beetle *Cathartus quadricollis* carried towards the store fungi belonging to *Penicillium* sp., *Aspergillus* sp., and *Fusarium* spp [5]. Likewise, Khan et al. [53] reported that the insects *Sitotroga cerealella*, *Tribolium castaneum,* and *Rhyzopertha dominica* enabled the propagation of the mycotoxigenic fungi *Alternaria alternata*, *Alternaria infectoria*, *Aspergillus flavus*, and *Aspergillus tenuissimain* in stored wheat grains. On the other hand, no significant differences were found in ergosterol content between treatments with and without weevils in silo-bags containing fungi and sulcatone. A possible explanation for these results may be that sulcatone could be exerting its insecticidal effect at an early stage of the experiments, so insects were not allowed to act as vectors. Regarding mycotoxins, the presence of the maize weevil increased fumonisin B_1_ biosynthesis in an indirect manner, by increasing fungal dispersion and biomass, which was consistent with results from Ferreira-Castro et al. [51]. However, as fumonisin B_1_ results were expressed as the amount of fumonisin B_1_ in relation to ergosterol content, no significant differences were observed between the silo-bags containing insects and those without insects (Table 2). 

On the other hand, the silo-bag experiment set out to evaluate the potential application of sulcatone as a natural VOC for the integrated control of the maize weevils and maize phytopathogenic fungi in a stored grain system. The insecticidal activity of sulcatone toward *S. zeamais* has already been discussed in previous paragraphs. As presented in Table 2, insect mortality was significantly higher in silo-bags containing *F. verticillioides* + *A. flavus* + *A. parasiticus + S. zeamais* + sulcatone, with 85.09 ± 1.46% mortality, while silo-bags with *S. zeamais +* sulcatone (without any fungal species) presented a significantly lower mortality of 71.69 ± 1.57%. This is a rather remarkable outcome since the presence of the fungal species contributes to the mortality of insects. In fact, silo-bags containing the three fungal species and the maize weevil (without sulcatone) showed a mortality value of 32.24 ± 3.68%, significantly higher than that of silo-bags only with insects (without sulcatone and fungi). These results evidenced that certain fungal metabolites might be exerting a toxic effect on *S. zeamais*. In this context, the exposure of *S. zeamais* to a fumonisin B_1_ extract showed repellency, yet there was no effect on insect mortality, which suggests that other fungal metabolites might be involved [54]. A possible explanation might be the presence of certain fungal VOCs since their insecticidal effect on *S. zeamais* and other species of insects has been documented in the literature [24,31,55]. Fungal VOCs are synthesized as side- and end-products during the primary and secondary metabolism [56]. The filamentous fungi *F. verticillioides*, *A. flavus*, and *A. parasiticus* are known to be prolific producers of VOCs [25,57,58,59,60,61,62,63]. On the other hand, Table 2 compares fungal biomass and fumonisin B_1_ biosynthesis in silo-bags containing the three fungal species alone and silo-bags containing the three fungal species with sulcatone. Both ergosterol content and fumonisin B_1_ biosynthesis were significantly reduced by 60% when sulcatone was added to the silo-bags. The antifungal effect observed in the silo-bag experiment was consistent with the results from the experiments conducted in Petri plates. However, the antifungal effect of the natural VOCs is not necessarily related to the antimycotoxigenic activity since mycotoxin production can be inhibited without fungal growth being affected and vice versa. In the present study, the amount of fumonisin B_1_ was normalized to ergosterol content to discriminate if a lower value of fumonisin B_1_ was due to an impairment in its biosynthesis or a weaker fungal growth. Data from silo-bag experiments showed that the amount of fumonisin B_1_ was significantly reduced by more than 60% in the presence of sulcatone. Previous studies reported a reduction in fumonisin B_1_ biosynthesis with trans-2-hexen-1-ol, 1-octyn-3-ol, and ethyl 3-methylbutanoate in maize grain bioassays [26,28,57]. Additionally, the antimycotoxigenic effect of ketones was previously reported for 3-hexanone and 4-hexen-3-one, with the latter being the most effective with 82% inhibition of fumonisin B_1_ with respect to the control [25]. 

The overuse of synthetic pesticides along with their high stability has led to their accumulation in soils, which represents a serious problem since they come into contact with seeds, being able to impair seed germination of feed crops [64]. In this context, even though natural VOCs are biodegradable, many of them were reported as toxic to different seeds when applied as fumigants, either by inhibiting germination or reducing stem and root length in a dose-dependent manner [24,65,66]. For these reasons, to ensure the safety of sulcatone as a pesticidal compound, its phytotoxicity on maize grains was evaluated. As displayed in Table 3, sulcatone showed no significant effect on the germination of maize seeds at any of the concentrations evaluated, which highlights the potential of this compound to be used against *S. zeamais* and its associated fungal species in a maize storage system.

## 4. Conclusions

Stored maize is frequently attacked by different pests, such as the insect *S. zeamais* and the phytopathogenic fungi *F. verticillioides*, *A. flavus*, and *A. parasiticus*. Synthetic insecticides and fungicides are currently used for their control, yet the interaction among pests ultimately determines the quality of grains in the silos and is usually ignored. The present study demonstrated that *S. zeamais* promotes fungal infection in maize grains. The maize weevil was involved in a significant rise in fungal biomass and fumonisin B_1_ biosynthesis, either through their feeding habits or their constant movement, acting as a vector of fungi and mycotoxins. On the other hand, the increasing tendency for safer food commodities demands environmentally friendly pest control agents. In this context, the natural VOC sulcatone proved to be effective against the maize weevil and the filamentous fungi. The strong repellent, insecticidal, fungicidal, and antimycotoxigenic effects reported and discussed in the present study, in addition to its lack of phytotoxicity on grains, highlights the potential of sulcatone to be used in a botanical-based pesticide for the combined control of insects and fungi in stored maize. 

## Figures and Tables

**Table 1:** Antifungal activity of sulcatone against *F. verticillioides*, *A parasiticus*, and *A. flavus*.

Fungal Species	Concentration (mM)									
	MIC ^b^	0 (Control)	0.03	0.06	0.13	0.27	0.54	1.06	2.12	4.24
*F. verticillioides*	3.5	0.0 ± 0.0	23.5 ± 0.0	29.1 ± 0.3	46.3 ± 0.4	56.0 ± 0.6	63.4 ± 0.6	75.2 ± 0.4	87.2 ± 0.6	100.0 ± 0.0
*A. parasiticus*	3.8	0.0 ± 0.0	1.3 ± 0.4	8.4 ± 0.0	12.9 ± 0.6	20.0 ± 0.4	35.9 ± 1.1	48.5 ± 0.7	71.2 ± 0.8	100.0 ± 0.0
*A. flavus*	3.9	0.0 ± 0.0	0.0 ± 0.0	0.0 ± 0.0	1.5 ± 0.2	13.9 ± 1.0	30.0 ± 0.6	41.3 ± 0.5	60.2 ± 0.7	100.0 ± 0.0

Inhibition of fungal growth determined after 7 days of exposure to sulcatone. Values represent means of inhibition percentage ± standard error (SE); The experiments were performed twice in quintuplicate. ^b^ Minimal inhibitory concentration (mM). MIC values were calculated through a linear regression and the following equations were obtained: *F. verticillioides*: y = 15.8x + 43.4; *A. parasiticus*: y = 22.5x + 13.6; *A. flavus:* y = 23.6x + 5.9.

**Table 2:** Ergosterol, fumonisin B_1_, and insect mortality in different silo-bag treatments.

Silo-Bag Treatment	Ergosterol (µg/g Maize)	Fumonisin B_1_ (µg FB_1_/ µg Ergosterol)	Mortality of *S. zeamais* (%)
*F. verticillioides* + *A. flavus* + *A. parasiticus*	100.11 ± 5.60 (c)	0.086 ± 0.015 (c)	-
*F. verticillioides* + *A. flavus* + *A. parasiticus + S. zeamais*	142.18 ± 8.82 (d)	0.073 ± 0.014 (c)	32.24 ± 3.68 (b)
*F. verticillioides* + *A. flavus* + *A. parasiticus +* sulcatone	41.82 ± 3.54 (b)	0.032 ± 0.08 (b)	-
*F. verticillioides* + *A. flavus* + *A. parasiticus + S. zeamais* + sulcatone	42.42 ± 5.29 (b)	0.036 ± 0.03 (b)	85.09 ± 1.46 (c)
*S. zeamais*	17.88 ± 0.77 (a)	0.00 ± 0.00 (a)	8.76 ± 2.89 (a)
*S. zeamais +* sulcatone	16.51 ± 1.59 (a)	0.00 ± 0.00 (a)	71.69 ± 1.57 (b)
Control (maize)	9.87 ± 0.75 (a)	0.00 ± 0.00 (a)	-

Values represent mean ± SE. Within each column, means with different letters indicate significant differences according to a DGC a posteriori test (*p* < 0.05). The concentration of sulcatone used in the experiment corresponded to 2 × 3.9 mM, the higher MIC value estimated for the three fungal species.

**Table 3:** Seed vigor (%) of maize grains treated with sulcatone at different concentrations.

Concentration	Seed Vigour (%)
Control	100 ± 0 (a)
LC_95_	94.9 ± 0.3 (a)
Repellent	91.5 ± 10.6 (a)
2 × MIC	79.7 ± 1.0 (a)

Values represent mean ± SE. Means with the same letter indicate non-significant differences according to a DGC a posteriori test (*p* < 0.05). Seed vigor was calculated through the equation *seed vigor* = ∑(*n d*^−1^) where *n* is number of seeds germinated at each day and *d* is the number of days elapsed since the beginning of the experiment.

## Data Availability

The original contributions presented in the study are included in the article, further inquiries can be directed to the corresponding author.
